# Ethical and social reflections on the proposed European Health Data Space

**DOI:** 10.1038/s41431-024-01543-9

**Published:** 2024-02-14

**Authors:** Ciara Staunton, Mahsa Shabani, Deborah Mascalzoni, Signe Mežinska, Santa Slokenberga

**Affiliations:** 1 Institute for Biomedicine, Eurac Research, Bolzano, Italy; 2 School of Law, University of Kwazulunatal, Durban, South Africa; 3 Faculty of Law and Criminology, Ghent University, Gent, Belgium; 4 Department of Public Health and Caring Science, Uppsala University, CRB, P.O. Box 256, 751 05 Uppsala, Sweden; 5 Institute of Clinical and Preventive Medicine, University of Latvia, Riga, Latvia; 6 Faculty of Law, Uppsala University, Uppsala, Sweden

**Keywords:** Ethics, Social sciences

## Abstract

The COVID-19 pandemic demonstrated the benefits of international data sharing. Data sharing enabled the health care policy makers to make decisions based on real-time data, it enabled the tracking of the virus, and importantly it enabled the development of vaccines that were crucial to mitigating the impact of the virus. This data sharing is not the norm as data sharing needs to navigate complex ethical and legal rules, and in particular, the fragmented application of the General Data Protection Regulation (GDPR). The introduction of the draft regulation for a European Health Data Space (EHDS) in May 2022 seeks to address some of these legal issues. If passed, it will create an obligation to share electronic health data for certain secondary purposes. While there is a clear need to address the legal complexities involved with data sharing, it is critical that any proposed reforms are in line with ethical principles and the expectations of the data subjects. In this paper we offer a critique of the EHDS and offer some recommendations for this evolving regulatory space.

## Introduction

As we emerge from the COVID-19 pandemic, the benefits of global data sharing to address a global pandemic are evident. Data sharing enabled health care policy makers to make decisions based on real-time data, it enabled the tracking of the virus, and importantly it enabled the development of vaccines that were crucial to mitigating the impact of the virus. This could only have been achieved through local, national, and crucially international data sharing. This global data sharing, however, was the exception and not the norm. Efforts to share data for research are challenging due to legal and ethical issues that include privacy, personal data protection, consent, risk of discrimination and stigmatization [1–3]. In recent years, researchers have cited the General Data Protection Regulation (GDPR) as hampering the sharing of personal data for research [4]. This is in part due to the fragmented implementation of the GDPR across the European Union (EU) Member States and lack of clarity in legal bases for secondary uses of data [5, 6].

In May 2022, the draft regulation for the European Health Data Space (EHDS) was published. As part of the European Commission’s plans to build a strong European Health Union and to realize the potential that data holds for the economy [7], the draft regulation is proposing one legal framework to facilitate access to electronic health data across all Member States for eight specified purposes that, as described in Recital 1 “would benefit society such as research, innovation, policy-making, patient safety, personalized medicine, official statistics or regulatory activities”. The aim is that this will address some of the elements of the GDPR that are perceived to hamper data sharing [4]. The ambition is that enabling access to data will improve research, policy making, and innovation, leaving the EU and Member States better able to respond to health crises and future pandemics, improve treatment and care, and increase the competitiveness of the EU, amongst other purposes.

A framework that facilitates data sharing is without a doubt needed. Such a framework must provide for data sharing through processes that respect the preferences and values of participants, have oversight mechanisms in place, and processes to ensure the equitable benefits arising from such data sharing. This data sharing framework also needs to account for the multitude of risks that arise in the secondary use of data. This may be due to the risks associated with the type of data itself (e.g., there are greater risks associated with the use of genetic data), but also risks associated with data use (e.g., data can discriminate certain groups or populations based on prevalence of a condition or if they have certain characteristics that correlates with unhealthy conditions). Concerns such as stigmatization, discrimination, exploitation, and other data driven harms are often contextual depending on the use, including the entity using that data, and a framework on data sharing must have processes in place to account for these concerns.

The EHDS is a real opportunity to introduce such a framework. A legally sound and ethically robust data sharing process for secondary use should result in a social licence for the secondary use of data [8]. A social licence refers to the fulfilling expectations of society in certain activities that go beyond the requirements set out in formal regulation [8, 9]. In the context of the secondary use of data, this would require not only compliance with the applicable laws, but also ensuring that the use of this data is in line with the expectations of society. However, from the draft EHDS text, it does not appear that sufficient consideration has been given to the ethical, legal and social issues (ELSI) that must be addressed in data sharing [1, 10–14]. The secondary use framework is a data access regime put forward by the European Commission that is anchored in the GDPR model and the right to data protection. Data protection is critical but only one issue to be considered in data sharing. Other factors, specifically the right to autonomy, a right that in the context of data driven methods relates to the right to decide over what will be done with ones’ own data, and the need to guard against discrimination and stigmatization that can occur on a group level must be considered, in addition to the potential benefits that can accrue in benefit sharing.

Changes to the draft EHDS text that are to be proposed by the European Parliament in the legislative process address to some degree issues related to the right to autonomy (e.g., the proposed inclusion of an opt-out provision for those who do not wish their data to be shared in certain contexts), but do not address the wider ELSI concerns in this domain. At this time, there is a potential for improvements to the text and the purpose of this paper is to offer some critiques and propose solutions. In this paper, when discussing the draft EHDS, we refer to the draft text and the proposed amendments, unless otherwise stated. We first briefly discuss the current ethical processes in the secondary use of data and the changes brought about by the introduction of the GDPR. Second, we set out the EHDS and the proposed framework on the secondary use of electronic health data. We then turn to critically reflect on the proposed framework demonstrating the problems in three areas: risks to natural persons, restriction of right to autonomy, the lack of ELSI informed approach to two new proposed processes. We conclude by offering proposals on how a revised regulation can enable an ethical approach to the secondary use of data under the EHDS.

## Ethical oversight and the secondary use of data under the relevant ethics guidelines and the GDPR

Research ethics is an important mechanism to balance the protection of the rights of research participants with the public value of science for society. Informed consent is a critical component of the ethical conduct of human subject research, but equally so is research ethics committee (REC) oversight. The REC provides an independent review to help ensure that research is scientifically and ethically sound with a fair balance between the risks to the participants and the benefit to society [15]. Informed consent and independent ethical review continue to be followed in the context of interventional research, but the development of data driven research methods has brought about changes to some of the procedures and governance of research ethics, as they have emerged from the Nuremberg Code, the Declaration of Helsinki, and the Belmont Report [16–18]. The reason for this change is that the risk to individual in data driven research is different. Interventional research carries physical risk, but the risks in the use of data for research relate to personal autonomy, privacy, and data protection. Beyond the individual, there is a risk that the use of data can lead to group level discrimination and stigmatization, due to the presence of certain conditions but also the correlation of characteristics with certain behavior [19, 20].

Data, however, can be collected, stored, used, and re-used with minimal *physical* risk to participants. Due to this minimal physical risk to participants, there have been changes in some of our long-standing ethical rules, particularly in relation to informed consent. These new research avenues have also identified new ethical challenges such as the return of results and incidental findings [21–27].

In the context of data driven research methods, we have seen a push to move from a paradigm that gives specific informed consent to one study, to broad consent that provides consent to certain unspecified research [28]. Due to concerns that broad consent is not informed consent, tiered consent that provides participants with a range of consenting preferences, and dynamic consent which uses information technology to facilitate consent to projects, change, and up-date their consenting preferences have emerged as potential new forms of consent for data intensive research [11, 23, 29, 30]. In most cases, consent alone is insufficient to protect participants and new governance models have emerged. For example, data access committees (DACs), are mechanisms through which data access requests are considered and can  balance the competing rights and interests of the participants, the data holder, and the interests of science [13, 31, 32]. In light of these new ethical challenges and governance processes, new guidance has been developed for data intensive research methods, such as the Declaration of Taipei which evolved from the Declaration of Helsinki [33].

Perhaps the most significant change in the regulation of data intensive research methods, has been the introduction of the GDPR. Introduced to harmonize rules and principles on the protection of personal data across the EU, key principles must now be met in the processing of personal data and data subjects have certain rights in the processing of their personal data. In recognition of the importance of scientific research, the GDPR provides for certain exceptions or derogations for research [5]. While often the primary concern in the regulation of data intensive research methods, the GDPR is not a research regulatory instrument. Recital 33 makes reference to “recognized ethical standards for scientific research” in the context of consent, but beyond this, the GDPR does not take account of or integrate other ethical norms, standards and practices. The purpose of GDPR is the protection and free movement of personal data thus the GDPR does not account for the other ethical concerns and challenges that need to be addressed in data intensive research.

When collecting data from individuals for the first time for research, the principles of research ethics require REC approval and informed consent. Under the GDPR, data subjects have the right to be informed about aspects of the use of their data, including the purposes for which the data can be used. This right ensures transparency and provides data subjects with the right to object to the use of their data in research. This is not always the case for the secondary use of data. Due to derogations provided for research under the GDPR, the right to information can be derogated from if the personal data is not collected directly from the participant. If a participant is not aware that their data is being processed for research, they are not able to exercise their right to object, leaving a participant with a limited ability to exercise their autonomous choice in what their data will be used for [34].

Despite the legal acceptability of the derogation of the right to information, there is considerable ELSI work that has looked at participants perspectives on the use of their data in research. While participants are often motivated by altruism to give their data for research and this should continue to be encouraged, they often have clear preferences on the use of their data and concerns that must be considered and addressed when their data is used for research [19, 35]. In other contexts, stigmatization and discrimination has emerged as concerns, particularly in the use of genetic and other health related data [19]. Thus it is important that there are checks in place to guard against data harms. It is for this reason that some of us have called for an integrated ethics approach to the interpretation and application of the GDPR for research by which we mean the safeguards required under Article 89 when personal data is being processed for research should be informed by safeguards required as part of the ethical conduct of research [36]. This could ensure that the primary and secondary use of personal data in research follows current ethical practices and principles of human rights that go beyond just data protection concerns in data intensive research methods.

## Accessing electronic health data for secondary purposes under the EHDS

In part response to the fragmented implementation of the GDPR, the proposed EHDS is seeking to create a *legal obligation* on data holders to share electronic health data for secondary purposes if certain conditions are met. Slokenberga has comprehensively detailed the process for accessing electronic health data for secondary purposes, but key elements are worth noting [37].

Any natural or legal person can apply for access to the electronic health data (called a “data user”) from a data holder (“any natural or legal person, which is an entity or a body in the health or care sector, or performing research in relation to these sectors”). Electronic health data is broadly defined and includes electronic health records, genetic data, and population-based health data. Most of the data that is available to be accessed, will by and large, be data that has been collected in the context of clinical care or research. The data user can use this data for one of the currently eight proposed secondary uses under Article 34(1) that includes public health activities in the public interest, scientific research related to the health or care sector, training and testing of AI, providing personalized care, amongst others.

There are currently two proposed avenues for accessing electronic health data. If access is sought from one data holder from one Member State, access can be applied directly to the data holder themselves under Article 49. For all other cases, access is decided by a Health Data Access Body (HDAB), a new entity to be established in each Member State. Under Article 36(1), a Member State may establish one or more new public sector bodies to fulfill the role, or rely on either an existing public sector bodies or an internal services of public sector bodies.

The many duties of the HDAB include approving data access applications, issuing data permits, process electronic health data for secondary purpose, including the collection, combination, preparation and disclosure of those data for secondary use on the basis of a data permit, and making public a national dataset catalog (Article 37). The HDAB must cooperate with bodies that includes supervisory authorities under the GDPR, and other stakeholders, including patient organizations, representatives from natural persons, health professionals, researchers, and ethical committees, where applicable in accordance with EU and national law.

An access application should include a detailed explanation of the purpose of the data use; description of the requested data; a justification why anonymous data would not suffice if access to pseudonymized data is sought; (undefined) safeguards to prevent unauthorized use and protect the rights and interests of the data holder and natural persons; estimated time period data is required; details on a secure processing environment. If personal data is requested, the applicant must describe how they comply with the GDPR and information on any ethical aspects that are applicable. Article 44 also makes it clear the importance of the principle of data minimization and purpose limitation in the HDAB’s assessment.

The HDAB must make an assessment within two months of receiving an application, extendable by two months for complex applications. If the criteria are met, the HDAB requests the data from the data holder. The HDAB makes the data available to the data user through a secure processing environment within two months. A data permit is then issued to the data holder specifying the terms and conditions of the data use.

## Critical reflection of the EHDS and the proposed amendments through an ethical lens

The draft EHDS should enable the sharing of electronic health data for secondary purposes. Our concern is that the current proposed process would result in incursions on some of our ethical principles in research. Specifically we highlight four concerns: under the proposed EHDS data protection is the principle data harm that is considered; the special regime for research under the GDPR would apply to all stated purposes for secondary use under the EHDS; the proposed new process is a further erosion of the right to autonomy; and some of the purposed new processes need to be supported by ethical oversight and guidance before they are implemented.

## Assumption that data protection is the only concern in secondary use of data

The proposed EHDS is seeking to drive forward and streamline the data sharing agenda within Europe. It creates an obligation to share electronic health data, and Article 43 gives the HDABs the power to issue fines and penalties if data is not shared.

A reading of the EHDS would lead one to assume that data protection is the only concern in the secondary use of data. It establishes that, as a rule, the data should be released in an anonymized format unless pseudonymized data are necessary for the stated purpose. For pseudonymized data, the code key remains with the HDAB, and, as provided for in Article 44(3), any attempt by the data user to attempt reidentification “shall be subject to appropriate penalties”. In its application the data user must also describe safeguards to protect (amongst others) the rights and interests of the natural person (Article 45(2)(f)), without any consideration of what these rights are. Considering the tenor of the proposed regulation, it is most likely that this will be interpreted to mean rights and interests as they relate to data protection.

The European Data Protection Board (EDPB) and the European Data Protection Supervisor (EDPS) have issued a joint opinion critiquing the proposed regulation and demonstrating where, in their view, it does not conform the GDPR requirements [38]. We echo many of these critiques and do not restate them here. However, even if these critiques are addressed, they would not account for the inherent assumption throughout the proposed regulation that data protection is the only concern. Even if data is anonymized, the data can be misused, used beyond what an individual consented to or reasonably expected the data to be used for, and lead to group discrimination and stigmatization.

REC approval is one such mechanism that can guard against data misuse and other potential risks. It is for this reason that the Declaration of Taipei, the CIOMS guidelines and other ethical guidance, as well as some jurisdictions in Europe (e.g, Sweden) require REC review for the secondary use of data. The EHDS leaves the requirement of ethical assessment to Member States. Thus while the EHDS will introduce a harmonized legal framework that will streamline the *legal* process, but there is no such harmonization of ethical processes in the secondary use of data. Fragmentation will continue until both legal and ethical processes are harmonized across Member States.

Second, there is a duty on the data user *only* to assess the ethical aspects. If the data user is applying from a jurisdiction that is different to that of the data holder, differing ethical rules may apply. The data holder may be bound by ethical rules that restrict the sharing of the data without ethical review, but the EHDS does not provide an avenue for the data holder to object to the sharing of data on ethical grounds. For example, if a population biobank has obtained the consent of its participants to use the data for specified research purposes only, will they be forced to share the data for other purposes without going back to its population to recontact as per the consent? Without clarity, it is likely that this will create complex questions regarding the different ethical rules in each jurisdiction.

Third, ethical approval is generally only required where data is being accessed for research purposes. Thus, for data use that does not fall under research regulatory frameworks, national research ethics frameworks will not apply and no other avenue for potential oversight of these other potential data harms. Yet the concerns on the secondary use of data apply irrespective of whether the data is to be used for research, training and development of AI or other secondary purposes. This leaves us with a gap on how these ethical issues on data use should be addressed for data use outside of research going forward.

## Disproportionate extension of special research regime to other secondary purposes

The GDPR has provided for certain derogations for research, including derogation from the right to information. Without this right, data subjects will be unaware that their data is being processed and thus unable to exercise their other rights. However due to the importance of scientific research in society, a derogation to this right (and the associated downstream impact on other rights) is considered proportionate if the processing of the personal data is for research.

Under Article 38 of the proposed EHDS, the derogation to the right to information now applies to *all* proposed secondary purposes. Derogating from rights under the GDPR research regime can only be acceptable if certain conditions are met, determined on a case-by-case assessment. Under the proposed EHDS this derogation is now autonomatic [5]. The use of the data outside of research does not have any such protections in place such as REC oversight, nor is the derogation of the right to information to the seven other purposes balanced with any other standards or safeguards. While we welcome that Articles 38 and 39 mandate the Health Data Access Bodies to make available certain information to the public, this information is on a general level only and in no way remedies this wide extension of the derogation of right to information. Any extension of the erosion of data subjects’ rights must be justified with other mechanisms in place to ensure that the rights and interests of data subjects continue to be safeguarded.

## Consent, autonomy, trust, and social justice

Consent is one of a number of lawful bases for the processing of personal data and sensitive data under the GDPR. Some jurisdictions, such as Italy, have taken a restrictive approach to the implementation of the GDPR for research and require consent as the lawful basis for processing of genetic data for research. In other countries consent may not be mandated as the lawful basis of the processing of data, but required as part of the national ethical rules [39].

The draft EHDS is seeking to address this fragmented approach to the requirement of consent as a lawful basis and under Article 33(5) if consent is required by national law, “health data access bodies shall rely on the obligations laid down in this Chapter to provide access to electronic health data”. In the explanatory memorandum, this is justified on the basis that it is replacing it with “with a trusted governance and at lower costs than relying on consent”.

An appropriate consent process is an ongoing challenge for the data driven research community. Risks relate to privacy, stigmatization, and discrimination, but the potential use of data, particularly genetic data, impacts the individual, their family, community, and wider population from which the individual comes from. There is also a risk if appropriate governance processes are not put in place and data cannot be reused. Governance processes also need to account for benefit, particularly ensuring equity of benefit.

New governance models, particularly models that are premised on participatory governance, have been proposed. Prainsack’s model of solidarity is advocating for governance models whereby there are equitable harms and benefits across society and we share our data on this premise [20, 40–42]. Data trusts have been proposed whereby data subjects give their data to be managed by data trustees. The trustees are then responsible for stewarding the data for the benefit of others, known as the beneficiaries [43, 44]. While the benefit may not be for the data subjects themselves (and so would speak to data driven research methods), the data subjects decide on their involvement in the data trust and that there is ongoing stakeholder and public engagement [45]. Other proposed governance models include data commons and data cooperatives [46–48]. The legal framework proposed under the EHDS is thus somewhat similar to these proposals in that it is seeking to enable the secondary use of data. There are two notable differences: first, as discussed, the measures under the EHDS focus exclusively on the individual data protection concerns, and second is the treatment of consent under the EHDS.

Despite changes to the *process* of consent, consent remains a fundamental requirement in research ethics and in these other proposed models, the data subject does have a role in deciding on whether their data is to be part of the data trust (for example). Our concern is that in the original proposed text, the Commission is considering the current model of informed consent only, a model that is indeed burdensome, costly, and challenging to continuously go back and ask for consent. Broad consent and tiered consent while suitable options, do require re-consent if the purpose is beyond that what is specified in the consent, a process that may frustrate the data users as the technology and potential data use constantly changes. What is needed is a consent that is not static but can change in accordance with the data subject’s preferences and also potential data uses. What is not needed are legal frameworks that provide access to data without consent.

The UK and Australia are examples where there was a public push back to schemes that sought to share data without the consent of the patients [8]. Such proposed schemes failed for several reasons including poor public engagement, but they serve as a stark reminder that a social licence and public acceptance to access data for research cannot be assumed simply by having a legal framework in place. The EHDS is undoubtedly introducing new governance process for the secondary use of electronic health data. Public trust is also critical, but legal legitimacy alone does not automatically equate to trusted governance [49–51]. Lessons must be learned from the ELSI work in this domain to consider how it can become a trusted governance. Empirical research has demonstrated the importance of ongoing information about data use, and that individuals have preference on how their data will be used, preferences that must be respected.

This is of particular importance when one considers that under the proposed EHDS, data can be used for purposes that the individual did not consent, nor would have expected their data to be used. Once passed, a general information campaign should alert patients that their electronic health record may be used for secondary purposes. However, at the time that they provided their data, a patient is unlikely to have expected that their data collected as part of their individual electronic health record will be used for the development and training of AI, for example. Equally participants who have donated their data to a biobank, will expect that their data will only be used in accordance with that consent. Undoubtedly, they would not expect that their data will be used for purposes other than research. The EDPB/EDPS Joint Opinion on the EHDS has pointed out that this provision on consent is incompatible if consent has been the ground for the lawful processing of data under the GDPR. However, this issue extends beyond a conflict of laws situation as it could impact trust. Indeed, we would have concerns about the impact this could have on participant trust in biobank research if a biobank, for example, is obliged to share data for secondary purposes that go beyond the participants’ consent. The reputational damage and loss of trusted position that likely have in their community could be untold.

The draft Report from the European Parliament raises this point and draft amendment 13 recommends that patients can opt-out to the secondary use of their electronic health data and to inform them of this possibility to opt-out. This is expanded further in draft amendment 84 that states:

“Natural persons that are subjects to secondary use of health data shall have the right to decline the processing of their health data. Health data access bodies shall provide for an accessible and easily understandable opt-out mechanism, whereby natural persons must be offered the possibility to explicitly express their wish not to have all or part of their personal electronic health data processed for some or all secondary use purposes. In situation where natural persons explicitly express their wish to use opt-out mechanism to data holders, data holders shall direct natural persons to the health data access bodies.”

We endorse this position sketched out by the European Parliament. This opt-out mechanism, if embedded in an appropriate thick information process [52] would provide a more equal balance between providing access to electronic health data for secondary purposes while also providing individuals with control over their electronic health data. It will also ensure that patients are empowered to have control over their electronic health data, which is one of the aims of the EHDS.

To realize this amendment, however, conversations now need to turn to how to manage this opt-out process. A system that facilitates communication with individuals to describe the purpose for which access to their data will be used and enable a response will be required. As an opt-out system is being envisaged, a dynamic interface that enables the provision of information to data subjects on data use and a process to enable them to actively opt-out will need to be developed. As this must be developed, we would also call for consideration to extend this provision beyond opt-out only to enable the selection of an opt-in process also for those who would wish to have to opt-in only i.e., the default that their data not be used unless they actively consent to the use of their data for the proposed purpose if they so wish. Let us pause to consider that for one moment. There have been discussions and considerations on whether citizens have an ethical duty to share their health information for research without consent [12, 53]. There have also been calls for solidarity (understood in this context as a wiliness to share data for the benefit of others) to underpin the governance of data driven research [40, 50]. However to realize solidarity-based approaches, the benefits and costs of the access to and use of data should be borne collectively. Under the draft EHDS, the focus is on access to the data only, with no consideration of access to the down-stream benefits in possible outputs of the use of the data. Can we really guarantee that any downstream benefits will be equally shared amongst citizens? Leaving the issue of benefit to one side, there is research demonstrating concerns about commercial bodies having access to data [35, 54, 55]. While we recongise the important role of industry in the development of therapeutics and treatments, citizens’ concerns must be adequately accounted for. Citizens who have concerns on data sharing may be frustrated that they constantly must opt-out of a system that they do not want to opt-in to, except in limited circumstances. If we are designing a system that provides for opt-out, it can also facilitate opt-in. The system could give citizens the possibility to select opt-in, opt-out, or indeed indicate that they are happy to share their data for any purposes that has been approved by the new governance process.

Increasingly there are examples of the application of dynamic consent and e-consent being integrated into research [23, 56]. Critiques of dynamic consent have raised concerns that there would be many withdrawals leading to high drop-out rates, that the system would be difficult to manage, particularly for those who are not digitally literate, or that the system is not scalable. Dynamic consent does require investment in the setting up of the system, both in terms of infrastructure and personnel. It also requires expertize in participant engagement and communication. This is a system, however, that will need to be built to support and manage the opt-out and can be adapted to support an opt-in. As to the concerns that it will lead to high dropout rates, this has not transpired in the experience of two of the authors of this paper in the practical application of dynamic consent in a population biobank [11]. Furthermore, high dropout rates would be more likely due to concerns around the data use or system and not the consent process. The solution to managing high drop out rates is not an erosion of the choice, but an unpacking of why there are such rates and identifying solutions to address those concerns. Participants will also need to be informed about their responsibilities for keeping their contact information up to date so that they can opt out or opt in (depending on their preferences). They should also be informed about the importance of the use of their data and the risks for development and innovation if their data is not use. In this way, discussions on consent, autonomy, opt-in, and opt-out, can be presented in the context of the role of their data in wider society.

It is worth noting that although the Parliament’s draft proposal addresses the issue of the right to autonomy of the individual, it is not clear whether Article 33(5) also applies when consent is required as part of national ethical requirements (as distinct from when consent is the lawful basis of processing). If the ethical assessment that may be required (depending on Member State law) as part of the secondary use of data requires the use of the data to be in line with consent, can this override the EHDS provisions on consent? Once again there is uncertainty in the interplay between national ethical rules and proposals under the EHDS.

## EHDS and the return of results

In addition to creating an obligation to share data, the draft EHDS introduces the possibility of return of results to individuals for data that is not anonymous. Article 46(12) provides that a condition of the data permit is that data users are to inform the HDAB of “clinically significant findings that may influence the health status of the natural persons whose data are included in the dataset”. We very much welcome this proposal as returning results to individuals that arise in the secondary use of their data signifies respect to the participant and enables the individual to be more fully informed about their health. However, this provision as currently framed in the EHDS is in its infancy and we currently see two major issues that needs to be addressed.

First, there is emerging ethical consensus that clinically significant and actionable research results should be returned to participants, although there is not always clear consensus on what clinically significant results means in research fields such as genetics and genomics and for various age groups. Under the current proposal, the decision on whether to return will rest with the HDAB alone. It is generally accepted that the return of results should be in line with the preferences of the participants [27]. Yet, under the current proposal, there is no role for the individual in the decision making process on what and when to return. The data user must share the results to the HDAB, and it is this body that will decide on whether results shall be returned. This brings the danger that differences in approach will arise between health data access bodies and whether individuals obtain clinically relevant information could depend on the relevant HDAB.

Second, for those health data access bodies who decide to require the return of such data, this is a process that is fraught with ethical complexities and is not as simple as just informing the individual [26, 27, 57]. Numerous guidelines from research bodies and consortia have attempted to ethically manage this process and they generally centre on key principles that respects the autonomy of the participant and their right to know or not to know in a clear and transparent process. They may have preferences on the type of findings to be returned, but there is no place here for the individuals autonomous preference. It is critical that the person returning the results has the necessary training and skills to understand, communicate, and explain the results and their impact. This is a process that should be included in the final EHDS, but it is critical that it is supported by ethical best practice.

## Recommendations on the way forward: enabling an ethical approach to the secondary use of data

We very much welcome the move towards furthering data sharing. Data sharing for certain secondary purposes will likely have an important role in protecting and promoting a healthy population. However, it must be done in a manner that understands and actively works to maintain the social licence and public trust in the secondary use of data. This is particularly critical in the context of the EHDS as the electronic health data that it is anticipated will be shared come from areas traditionally vested in high levels of trust: healthcare and scientific research. Any system that creates a legal obligation to share data must be crafted in a way that does not risk undermining trust. Loss of trust can have far-reaching implications for medical care, deterring people from disclosing important information. Loss of trust in scientific research risks impairing willingness to participate in research, ultimately negatively affect scientific research, and risks upending one of the stated purposes of the EHDS. Currently there is insufficient regard to individual rights and interests, beyond some data protection considerations, but the proposals do not also consider the risks beyond the individual. It must be remembered that while data sharing may be beneficial and bring about public benefit in the form of therapies or new treatments, data can also be used to stigmatize and discriminate [19]. Furthermore, simply because a benefit is available it does not follow necessarily that there is a fair distribution of benefits resulting from access to data. Data sharing during the COVID pandemic did indeed lead to the quick development of vaccines, but the reality is that once the vaccines were developed economic interests and not need or public benefit determined access [58–61].

We argue that the framework on the secondary use of data proposed under the EHDS is contingent on an ethically sound approach that protects the rights of participants and has oversight mechanisms in place to mitigate against data harms beyond data protection. Such an approach would ensure that public trust is maintained. To achieve this, there is first the need to shift the power asymmetry. Under the initial proposal, the power asymmetry between the data subject and those deciding on their data access has widened to the point that the individual has no power. There is scope to change this by incorporating some of the Parliament’s draft recommendations. Processes and ultimately technical infrastructure are needed to support the Parliament’s draft proposals and the solution may be found in Chapter III. Chapter III is seeking to build a system of interoperable electronic health records system. This will be transformative and enable individuals to exercise more control over their health data, but it currently applies to primary use (i.e., healthcare) only. There is a real opportunity now to incorporate the secondary use of data as part of this system and develop an interactive and dynamic infrastructure. Such a system can enable individuals to express their consenting preferences, change them, and withdraw. It can also ensure transparency and enable individuals to be informed, thus preserving their right to information and ability to exercise their other rights.

Second, Article 36(3) and Article 37(2)(c) provides that health data access bodies should cooperate with stakeholders that includes patient organizations, researchers, and ethics committees in the exercise of their tasks, but the level of cooperation or engagement is unclear. We argue that this should be a requirement to involve patient representatives and ethics experts in the composition of health data access bodies. This can ensure that there is ethical oversight and reflection as part of the process and would also be a more participatory form of governance. Health data access bodies should also have the power to mandate when they consider it necessary to seek REC approval as part of a data access request process.

Third, a revised draft needs to account for the additional ELSI concerns that go beyond data protection and re-identifiability in the secondary use of data and consider how these concerns can be addressed in the secondary use of data. Importantly the right to autonomy, guarding against discrimination and stigmatization for the individual as well as the risks to others in the use of data. Equally if it is introducing a proposal on the return of results, this should be developed in line with emerging ethical consensus on how these results should be returned.

Finally, the reality of the proposals needs to be fully understood. One legal framework for the secondary use of electronic health data will bring some clarity, but differences in ethical rules remain at a Member State level. Currently it is unclear what will occur when the proposals under the EHDS and national ethical rules are in conflict. Guidance on how to navigate these issues will be needed, otherwise confusion could result in a deadlock in accessing data, and the EHDS may not be operationalizable.

Changes to resolve these issues are needed to make the dream of the EHDS a reality. In its revision we call for a legal framework that enables access to data for secondary use in a manner that balances the rights and interests of *all* stakeholders. An appropriate consent process is only one element in this governance framework, but it is a process that must be maintained.
